# Retrospective Evaluation of the Prevalence and Prognosis of Hypochloremia in Dogs and Cats

**DOI:** 10.1111/vec.70053

**Published:** 2025-11-19

**Authors:** Yu Ueda, Steven E. Epstein, Kate Hopper

**Affiliations:** ^1^ Department of Clinical Sciences, College of Veterinary Medicine North Carolina State University Raleigh North Carolina USA; ^2^ Department of Surgical and Radiological Sciences, School of Veterinary Medicine University of California, Davis Davis California USA

**Keywords:** acid–base balance, chloride, electrolytes, metabolic alkalosis, water balance

## Abstract

**Objective:**

To determine the prevalence, case‐fatality rate, and primary disease processes associated with corrected hypochloremia (hypo[Cl^−^]) in dogs and cats.

**Design:**

Single‐center retrospective study.

**Setting:**

Electronic medical records were reviewed to identify dogs and cats with at least one chloride and sodium concentration measured simultaneously during a 60‐month period.

**Animals:**

A total of 17,120 dogs and 4197 cats presented to a veterinary teaching hospital.

**Interventions:**

None.

**Measurements and Main Results:**

Measured hypo[Cl^−^] was diagnosed in 23.3% (3981/17,120) dogs and 59.0% (2475/4197) cats. Corrected hypo[Cl^−^] was diagnosed in 13.9% (2388/17,120) dogs and 34.9% (1463/4197) cats. The case‐fatality rates were higher in animals with measured and corrected hypo[Cl^−^] than those with normal corrected [Cl^−^] (*p* < 0.0001). The case‐fatality rate was also higher in cats with corrected hypo[Cl^−^] than those with measured hypo[Cl^−^] (*p* = 0.0002), but they were not different in dogs (*p* = 0.74). Of the dogs and cats with corrected hypo[Cl^−^], a total of 74.5% (1779/2388) dogs and 74.6% (1091/1463) cats were categorized as prehospital corrected hypo[Cl^−^], and a total of 20.9% (498/2388) dogs and 17.3% (253/1463) cats were categorized as hospital‐acquired corrected hypo[Cl^−^]. The case‐fatality rates of dogs and cats with hospital‐acquired corrected hypo[Cl^−^] were higher than those with prehospital corrected hypo[Cl^−^] (*p* < 0.0001). Various primary disease processes were identified in animals with corrected hypo[Cl^−^]. Of these, urologic, cardiovascular, and gastrointestinal diseases were the three most common disease processes identified in dogs and cats with corrected hypo[Cl^−^].

**Conclusions:**

Corrected hypo[Cl^−^] was a common electrolyte abnormality and was associated with higher case‐fatality rates than normal corrected [Cl^−^]. Various disease processes were associated with corrected hypo[Cl^−^], and closer attention to corrected hypo[Cl^−^] is warranted.

**Abbreviations**
CHF: congestive heart failure
CI: confidence interval
CPA: cardiopulmonary arrest
DKA: diabetic ketoacidosis
DM: diabetes mellitus
hypo[Cl^−^]: hypochloremia
MMVD: myxomatous mitral valve disease
OR: odds ratio

## 1 Introduction

Chloride ions play a crucial role in maintaining cellular functions and overall homeostasis in animals, encompassing serum electroneutrality, osmolality, acid‐base balance, hydrochloric acid production in the stomach, renal function, and various hormonal activity [1, 2]. Hypochloremia (hypo[Cl^−^]) is often observed in human patients with gastrointestinal, urologic, respiratory, and cardiovascular diseases [3, 4, 5, 6]. Similarly, hypo[Cl^−^] has been frequently documented in dogs and cats, with studies indicating its strong association with gastrointestinal diseases, congestive heart failure (CHF), and acute kidney injury [7, 8, 9, 10].

Measured hypo[Cl^−^] can result from loss of chloride ions and changes in free water balance [11, 12, 13]. When changes of measured chloride concentration ([Cl^−^]) are the result of free water balance alterations, sodium concentrations ([Na^+^]) also fluctuate proportionally. However, hypo[Cl^−^] could occur when [Cl^−^] is altered disproportionally to sodium ion changes [14, 15]. This condition may be induced by chloride ion wasting into the urinary or gastrointestinal system through fluid loss containing high [Cl^−^] and is described as corrected hypo[Cl^−^] [16, 17].

A retrospective study in cats revealed that approximately 27% of cats were diagnosed with measured hypo[Cl^−^], and 19% were diagnosed with corrected hypo[Cl^−^] [9]. In that study, common disorders associated with corrected hypo[Cl^−^] include gastrointestinal diseases, respiratory diseases, azotemia, and diabetes mellitus (DM). Another study reported that 39% of dogs with myxomatous mitral valve disease (MMVD) without signs of CHF developed measured hypo[Cl^−^], whereas 56% of dogs with MMVD with heart failure and 100% with refractory CHF had measured hypo[Cl^−^] [10]. In critically ill human patients with CHF, measured and corrected hypo[Cl^−^] were reported to be associated with an increased mortality rate [4, 12, 18, 19]. Recent studies in veterinary medicine have also reported a significant association between corrected hypo[Cl^−^] and an increased case‐fatality rate in both dogs and cats [20, 21].

Although previous research has shed light on the importance of hypo[Cl^−^] in both human and veterinary medicine, further investigation is warranted to better comprehend the underlying diseases and circumstances contributing to true chloride loss and corrected hypo[Cl^−^] in dogs and cats. The objective of the current study was to assess the epidemiology of corrected hypo[Cl^−^] in a large population of dogs and cats admitted to a tertiary referral hospital, primarily focusing on determining the prevalence, case‐fatality rate, and underlying disease conditions associated with corrected hypo[Cl^−^] in these animals.

## 2 Materials and Methods

### 2.1 Case Selection

The electronic medical records of the University of California, Davis, William R. Pritchard Veterinary Medical Teaching Hospital were searched for dogs and cats with measured serum or plasma chloride concentrations that were admitted between January 1, 2008, and December 31, 2012. Animals that had at least one whole blood or serum [Cl^−^] measured at admission or during hospitalization were further investigated. Animals were admitted through any service, including the emergency service or other specialty services, and those included in the study could be either systemically healthy or ill, provided that at least one simultaneous serum [Cl^−^] and [Na^+^] measurement was recorded at admission. The raw data containing first [Na^+^] and [Cl^−^], including 62,226 samples in dogs and 14,972 samples in cats, were extracted from the electronic medical record and transferred to a spreadsheet, along with patient signalment, final diagnosis, and outcome (survivor vs. nonsurvivor). Animals that did not survive, whether due to natural death or euthanasia, were categorized as “nonsurvivors,” while animals that survived and were discharged from the hospital were categorized as “survivors.” The raw data were visually inspected and manually corrected by removing the results obtained from other species or from nonblood samples. Patient data with missing or error values were also removed. Animals receiving potassium bromide as an anticonvulsant were manually removed from the dataset. The curated database included the time and date stamps of each measurement performed. Only the first of the multiple admissions to the hospital in an animal's lifetime was included if records showed multiple hospital admissions.

### 2.2 Data Collection

In each case, [Cl^−^] and [Na^+^] were measured using a serum biochemistry analyzer or a blood gas analyzer^1^. Heparinized whole blood samples were used for blood gas analysis immediately after sample collection. Blood samples collected in a red‐top tube were immediately submitted to the diagnostic laboratory for a serum biochemistry analysis^2^. Alternatively, the samples were centrifuged, and the supernatant serum samples were stored at 4°C until testing, which was performed within 12 h of sample collection.

The reference intervals of the measured [Cl^−^] and [Na^+^] for the blood gas analyzer and serum biochemical analyzer were previously generated based on samples obtained from clinically healthy dogs and cats. Animals with whole blood or serum measured [Cl^−^] lower than the reference intervals were identified and categorized as severe (≥10 mmol/L lower than the low end of the reference interval), moderate (7–9 mmol/L lower than the low end of the reference interval), mild (4–6 mmol/L lower than the low end of the reference interval), or borderline (≤3 mmol/L lower than the low end of the reference interval) measured hypo[Cl^−^]. No definitions for borderline, mild, moderate, or severe chloride disorders were identified in the veterinary literature, and the categorization used for the severity of hypo[Cl^−^] was chosen based on previous studies in both human and veterinary patients with minor modifications [22, 23, 24].

Corrected [Cl^−^] was calculated with the following formula based on simultaneously measured [Cl^−^] and [Na^+^] [16]:

$\mathrm{Corrected}[Cl^{-}]=\mathrm{measured}[Cl^{-}]\times \left(\mathrm{normal}[Na^{+}]/\mathrm{measured}[Na^{+}]\right).$

Measured [Na^+^] was not corrected for glucose concentration when calculating the corrected [Cl^−^]. The midpoint of the reference interval for measured [Na^+^] was used as the normal [Na^+^]. Using the formula above, the reference intervals calculated for plasma corrected [Cl^−^] were 110–121 mmol/L in dogs and 118–124 mmol/L in cats. These reference intervals were calculated based on plasma measured [Cl^−^] from the blood gas analyzer. The reference intervals for serum corrected [Cl^−^] were 108–116 mmol/L in dogs and 117–126 mmol/L in cats. These reference intervals were calculated based on serum measured [Cl^−^] from the serum biochemical analyzer. Animals with plasma or serum corrected [Cl^−^] lower than the reference intervals were identified and categorized as severe, moderate, mild, or borderline corrected hypo[Cl^−^], using the same criteria as for measured [Cl^−^]. The patient's outcome was extrapolated from the medical records.

Animals with corrected hypo[Cl^−^] were further evaluated to distinguish between prehospital and hospital‐acquired conditions, as well as primary disease processes, to assess the effects of chloride derangements independent of water balance changes. The timing of onset of corrected hypo[Cl^−^] was categorized as prehospital or hospital‐acquired. If corrected hypo[Cl^−^] was detected in the first blood sample at admission, it was categorized as prehospital corrected hypo[Cl^−^]. Animals with prehospital hypo[Cl^−^] did not require additional measurements of corrected [Cl^−^] during hospitalization. Hospital‐acquired corrected hypo[Cl^−^] refers to cases in which the initially obtained corrected [Cl^−^] was within the reference interval but subsequently fell below the reference interval during hospitalization. Although medical intervention typically does not start before the initial blood sample was obtained in our hospital, resuscitation or stabilization therapy may have been initiated beforehand and it was impossible to determine if animals received medical procedures before sample collection in this retrospective study. For dogs and cats with corrected hypo[Cl^−^] on admission that had received medical treatment by a referring veterinarian before being referred to our hospital, the timing of the onset of corrected hypo[Cl^−^] was considered unknown. If the corrected [Cl^−^] was normal at the initial measurement but corrected hypo[Cl^−^] was detected in the subsequent measurement during hospitalization, it was categorized as hospital‐acquired.

The medical records of animals with mild to severe corrected hypo[Cl^−^], whether prehospital or hospital‐acquired, were further reviewed to identify the primary disease processes, as reflected in the clinician's final diagnosis. The primary disease processes were categorized based on the major organ systems affected, including gastrointestinal, cardiovascular, respiratory, pancreatic, urologic, neurologic, hepatobiliary, oropharyngeal, musculoskeletal, reproductive, and hematologic [25]. The specific disease processes, including sepsis, toxicoses, neoplasia, hypoadrenocorticism, hyperadrenocorticism, hypothyroidism, hyperthyroidism, DM and diabetic ketoacidosis (DKA), diabetes insipidus, and cardiopulmonary arrest (CPA), were investigated individually. These conditions were investigated separately due to their potential multiorgan effects or their specific relevance to hypo[Cl^−^]. Animals could be counted more than once if they had more than one disease process potentially contributing to the development of corrected hypo[Cl^−^]. The prevalence of corrected hypo[Cl^−^] in relation to each specific disease process was determined by calculating the proportion of animals with corrected hypo[Cl^−^] among the total number of animals diagnosed with each primary disease condition. Animals with borderline corrected hypo[Cl^−^] were excluded from this analysis to create a clear distinction between animals with normal corrected [Cl^−^] and those with corrected hypo[Cl^−^].

### 2.3 Statistical Analysis

The outcome (survivor vs. nonsurvivor) of animals with or without measured or corrected hypo[Cl^−^] was compared with those with a normal measured or corrected [Cl^−^] using *χ* ^2^ analysis or Fisher exact test where appropriate. In addition, *χ* ^2^ analysis or Fisher exact test was used to compare the outcome of animals with measured hypo[Cl^−^] and animals with corrected hypo[Cl^−^]. The trend of the severity of measured hypo[Cl^−^] and corrected hypo[Cl^−^] and the case‐fatality rate were assessed with the Cochran–Armitage test for trend. In a post hoc analysis, the case‐fatality rates for borderline, mild, moderate, and severe corrected hypo[Cl^−^] were compared with those of animals with a corrected normal [Cl^−^] using *χ* ^2^ analysis. The numbers of animals with prehospital and hospital‐acquired corrected hypo[Cl^−^] were compared using *χ* ^2^ analysis or Fisher exact test. These same tests were used to analyze the association between the primary disease process and the documentation of corrected hypo[Cl^−^] in relation to each primary disease process. Bonferroni correction was applied to adjust for multiple comparisons of the primary disease processes. These statistical analyses were conducted using commercially available software^3^. A *p*‐value of <0.05 was considered significant (*p* < 0.002 after Bonferroni correction).

## 3 Results

**FIGURE 1:**
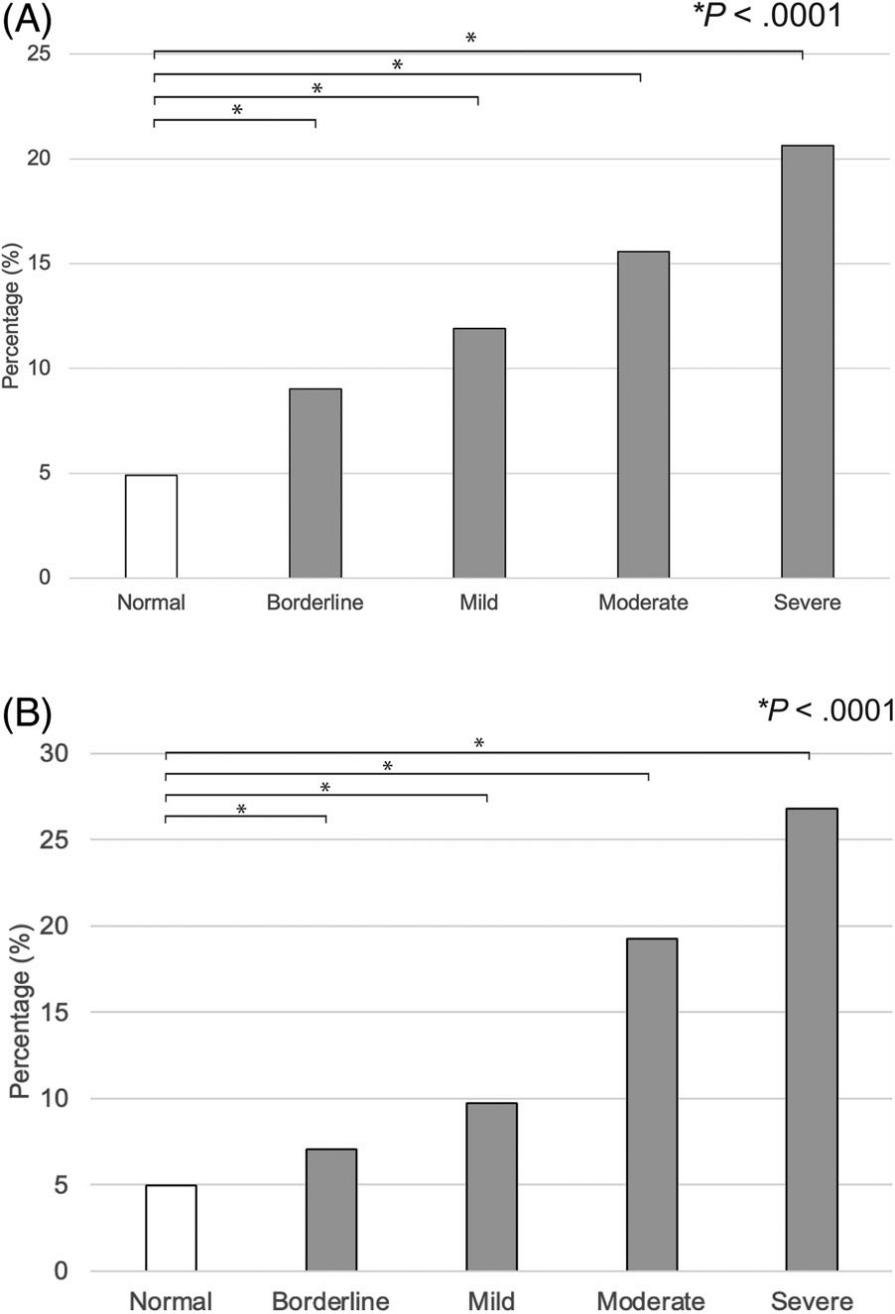
(A) Case‐fatality rates in 17,120 dogs with measured normal chloride concentration and measured hypochloremia. Significantly higher case‐fatality rates were observed in dogs with measured hypo[Cl^−^] compared with those with normal measured [Cl^−^] (*p* < 0.0001). (B) Case‐fatality rates in 4197 cats with measured normal chloride concentration and measured hypochloremia. Significantly higher case‐fatality rates were observed in cats with measured hypo[Cl^−^] compared with those with normal measured [Cl^−^] (*p* < 0.0001). Animals with blood or serum measured [Cl^−^] lower than the reference intervals were identified and categorized as severe (≥10 mmol/L lower than the low end of the reference interval), moderate (7–9 mmol/L lower than the low end of the reference interval), mild (4–6 mmol/L lower than the low end of the reference interval), or borderline (≤3 mmol/L lower than the low end of the reference interval) measured hypo[Cl^−^].

**FIGURE 2:**
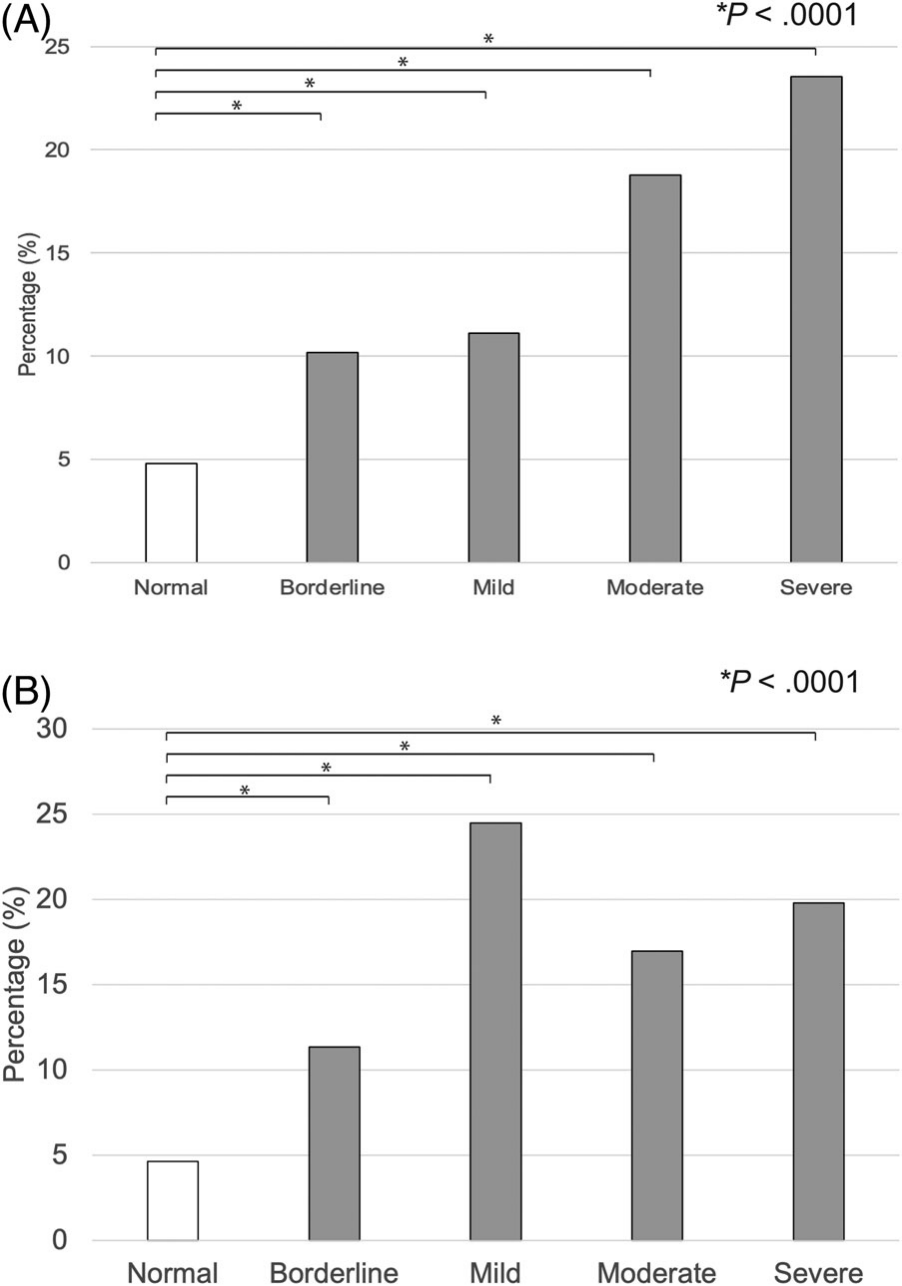
(A) Case‐fatality rates in 17,120 dogs with corrected normal chloride concentration and corrected hypochloremia. Significantly higher case‐fatality rates were observed in dogs with corrected hypo[Cl^−^] compared with those with normal corrected [Cl^−^] (*p* < 0.0001). (B) Case‐fatality rates in 4197 cats with corrected normal chloride concentration and corrected hypochloremia. Significantly higher case‐fatality rates were observed in cats with corrected hypo[Cl^−^] compared with those with normal corrected [Cl^−^] (*p* < 0.0001). Animals with blood or serum [Cl^−^]_corrected_ lower than the reference intervals were identified and categorized as severe (≥10 mmol/L lower than the low end of the reference interval), moderate (7–9 mmol/L lower than the low end of the reference interval), mild (4–6 mmol/L lower than the low end of the reference interval), or borderline (≤3 mmol/L lower than the low end of the reference interval) corrected hypo[Cl^−^].

During the 60‐month study period, raw data including 62,226 samples from dogs and 14,972 samples from cats were extracted from the electronic medical records. A total of 17,120 dogs and 4197 cats were ultimately included in the analysis, each with at least one simultaneous measurement of [Cl^−^] and [Na^+^] (Table 1). Of these animals, 58.7% (10,047/17,120) of dogs had normal measured [Cl^−^], while in the cat population, 31.6% (1326/4197) of cats had normal measured [Cl^−^]. In dogs with normal measured [Cl^−^], the case‐fatality rate was 4.9% (493/10,047) (Figure 1A), while in cats, it was 5.0% (66/ 1326) (Figure 1B). In dogs, 11,125/17,120 dogs (65.0%) had normal corrected [Cl^−^] values, while 2350/4197 (56.0%) cats had normal corrected [Cl^−^] values. Of the dogs with normal corrected [Cl^−^], a case‐fatality rate of 4.8% (535/11,125) was observed (Figure 2A), while the rate in cats was 4.6% (109/2350) (Figure 2B).

**TABLE 1:** Prevalence of normochloremia and hypochloremia in dogs and cats presented to a tertiary referral veterinary hospital over a 60‐month period.

	Dogs		Cats	
	Proportion (%)	Number (*n*)	Proportion (%)	Number (*n*)
All measured and corrected chloride		17,120		4197
Normal measured chloride	58.7	10,047/17,120	31.6	1326/4197
Normal corrected chloride	65.0	11,125/17,120	56.0	2350/4197
All measured hypochloremia	23.3	3981/17,120	59.0	2475/4197
Borderline	61.4	2446/3981	56.8	1407/2475
Mild	21.7	865/3981	23.7	586/2475
Moderate	8.1	321/3981	8.6	213/2475
Severe	8.8	349/3981	10.9	269/2475
All corrected hypochloremia	13.9	2388/17,120	34.9	1463/4197
Borderline	68.8	1642/2388	65.7	961/1463
Mild	20.7	495/2388	20.1	294/1463
Moderate	6.2	149/2388	7.7	112/1463
Severe	4.3	102/2388	6.6	96/1463

*Note*: Animals with blood or serum [Cl^−^] lower than the reference intervals were identified and categorized as severe (≥10 mmol/L lower than the low end of the reference interval), moderate (7–9 mmol/L lower than the low end of the reference interval), mild (4–6 mmol/L lower than the low end of the reference interval), or borderline (≤3 mmol/L lower than the low end of the reference interval) hypo[Cl^−^].

### 3.1 Measured Hypo[Cl^−^]

Measured hypo[Cl^−^] was found in 23.3% (3981/17,120) dogs and 59.0% (2475/ 4197) cats. Of these animals, 61.4% (2446/3981) dogs and 56.8% (1407/2475) cats exhibited borderline measured hypo[Cl^−^], while 21.7% (865/3981) dogs and 23.7% (586/2475) cats showed mild measured hypo[Cl^−^]. Moderate measured hypo[Cl^−^] was observed in 8.1% (321/3981) dogs and 8.6% (213/ 2475) cats, whereas severe measured hypo[Cl^−^] was found in 8.8% (349/3981) dogs and 10.9% (269/2475) cats (Table 1).

The overall case‐fatality rates in dogs and cats with measured hypo[Cl^−^] were 11.2% (446/3981) and 10.9% (269/2475), respectively. Specifically, in dogs, the case‐fatality rates were 9.0% (221/2446) for those with borderline measured hypo[Cl^−^], 11.9% (103/865) for mild measured hypo[Cl^−^], 15.6% (50/321) for moderate measured hypo[Cl^−^], and 20.6% (72/349) for severe measured hypo[Cl^−^] (Figure 1A). In cats, the case‐fatality rates were 7.0% (99/1407) for borderline measured hypo[Cl^−^], 9.7% (57/586) for mild measured hypo[Cl^−^], 19.2% (41/213) for moderate measured hypo[Cl^−^], and 26.8% (72/269) for severe measured hypo[Cl^−^] (Figure 1B).

Significantly higher case‐fatality rates were observed in both dogs and cats (*p* < 0.0001) with measured hypo[Cl^−^] compared with those with normal measured [Cl^−^]. The odds ratio (OR) for nonsurvival in animals with any degree of measured hypo[Cl^−^] was 3.67 (95% confidence interval [CI]: 3.22–4.18) in dogs and 5.45 (95% CI: 4.02–7.41) in cats. Furthermore, a linear association was found between the severity of measured hypo[Cl^−^] and higher fatality rates in both dogs (*p* < 0.0001) and cats (*p* = 0.0001). Post hoc analysis in dogs revealed that borderline (OR: 2.40 [95% CI: 2.04–2.82]; *p* < 0.0001), mild (OR: 3.71 [95% CI: 2.91–4.72]; *p* < 0.0001), moderate (OR: 12.3 [95% CI: 9.47–16.1]; *p* < 0.0001), and severe measured hypo[Cl^−^] (OR: 13.95 [95% CI: 10.14–19.20]; *p* < .0001) were associated with higher fatality rates compared with dogs with normal measured [Cl^−^]. Post hoc analysis in cats revealed that borderline (OR: 4.54 [95% CI: 3.12–6.54]; *p* < 0.0001), mild (OR: 5.36 [95% CI: 3.08–9.15]; *p* < 0.0001), moderate (OR: 9.42 [95% CI: 4.40–19.27]; *p* < 0.0001), and severe measured hypo[Cl^−^] (OR: 11.56 [95% CI: 4.80–25.82]; *p* < 0.0001) were associated with higher fatality rates compared with cats with normal measured [Cl^−^].

### 3.2 Corrected Hypo[Cl^−^]

Corrected hypo[Cl^−^] was diagnosed in 13.9% (2388/17,120) dogs and 34.9% (1463/4197) cats. Of these animals, 68.8% (1642/2388) dogs and 65.7% (961/1463) cats exhibited borderline corrected hypo[Cl^−^], while 20.7% (495/2388) dogs and 20.1% (294/1463) cats showed mild corrected hypo[Cl^−^]. Moderate corrected hypo[Cl^−^] was observed in 6.2% (149/2388) dogs and 7.7% (112/1463) cats, whereas severe corrected hypo[Cl^−^] was found in 4.3% (102/2388) dogs and 6.6% (96/1463) cats (Table 1).

The overall case‐fatality rates in dogs and cats with corrected hypo[Cl^−^] were 11.5% (274/2388) and 15.0% (219/1463), respectively. Specifically, in dogs, the case‐fatality rates were 10.2% (167/1642) for those with borderline corrected hypo[Cl^−^], 11.1% (55/495) for mild corrected hypo[Cl^−^], 18.8% (28/149) for moderate corrected hypo[Cl^−^], and 23.5% (24/102) for severe corrected hypo[Cl^−^] (Figure 2A). In cats, the case‐fatality rates were 11.3% (109/961) for borderline corrected hypo[Cl^−^], 24.5% (72/294) for mild corrected hypo[Cl^−^], 17.0% (19/112) for moderate corrected hypo[Cl^−^], and 19.8% (19/96) for severe corrected hypo[Cl^−^] (Figure 2B).

Significantly higher case‐fatality rates were observed in both dogs and cats (*p* < 0.0001) with corrected hypo[Cl^−^] compared with those with normal corrected [Cl^−^]. The OR for nonsurvival in animals with all degrees of corrected hypo[Cl^−^] was 2.62 (95% CI: 2.33–3.11) in dogs and 3.61 (95% CI: 2.84–4.66) in cats. Furthermore, a linear association was found between the severity of corrected hypo[Cl^−^] and higher fatality rates in both dogs (*p* < 0.0001) and cats (*p* = 0.0001). Post hoc analysis in dogs revealed that borderline (OR: 2.63 [95% CI: 1.99–3.47]; *p* < 0.0001), mild (OR: 6.67 [95% CI: 4.81–9.25]; *p* < 0.0001), moderate (OR: 4.27 [95% CI: 2.53–7.21]; *p* < 0.0001), and severe corrected hypo[Cl^−^] (OR: 5.15 [95% CI: 3.03–8.77]; *p* < 0.0001) were associated with higher fatality rates compared with animals with normal corrected [Cl^−^]. Post hoc analysis in cats also showed that borderline (OR: 2.63 [95% CI: 1.99–3.47]; *p* < 0.0001), mild (OR: 6.67 [95% CI: 4.81–9.25]; *p* < 0.0001), moderate (OR: 4.27 [95% CI: 2.53–7.21]; *p* < 0.0001), and severe corrected hypo[Cl^−^] (OR: 5.15 [95% CI: 3.03–8.77]; *p* < 0.0001) were associated with higher fatality rates compared with cats with normal corrected [Cl^−^].

### 3.3 Measured Versus Corrected Hypo[Cl^−^]

The case‐fatality rates of dogs with measured and corrected hypo[Cl^−^] were compared, and no difference was found (*p* = 0.74). There were also no differences in case‐fatality rates for borderline (*p* = 0.18), mild (*p* = 0.73), moderate (*p* = 0.42), and severe hypo[Cl^−^] (*p* = 0.58) when comparing measured and corrected hypo[Cl^−^].

In cats, the overall case‐fatality rate for corrected hypo[Cl^−^] was higher than that for measured hypo[Cl^−^] (*p* = 0.0002). The case‐fatality rates for cats with borderline (*p* = 0.0054), mild (*p* = 0.0001), and severe hypo[Cl^−^] (*p* = 0.031) were higher for corrected hypo[Cl^−^] compared with measured hypo[Cl^−^]. However, there was no difference in case‐fatality rates for cats with moderate hypo[Cl^−^], either measured or corrected (*p* = 0.65).

### 3.4 Prehospital Versus Hospital‐Acquired Corrected Hypo[Cl^−^]

Among animals diagnosed with corrected hypo[Cl^−^], 74.5% (1779/2388) dogs and 74.6% (1091/1463) cats were categorized as prehospital cases, while 20.9% (498/2388) dogs and 17.2% (253/1463) cats with corrected hypo[Cl^−^] were categorized as hospital‐acquired cases (Tables 2 and 3). A total of 4.6% (111/2388) dogs and 8.1% (119/1463) cats with corrected hypo[Cl^−^] at presentation received medical or surgical interventions by a referring veterinarian before admission to our hospital, and the time of onset of corrected hypo[Cl^−^] was thus reported as unknown for these cases. The overall case‐fatality rates with hospital‐acquired corrected hypo[Cl^−^] were higher than those with prehospital corrected hypo[Cl^−^] in both dogs (OR: 4.15 [95% CI: 3.18–6.76]; *p* < 0.0001) and cats (OR: 2.02 [95% CI: 1.44–2.84]; *p* < 0.0001) (Tables 2 and 3). When corrected hypo[Cl^−^] was categorized based on severity, the case‐fatality rate of dogs with hospital‐acquired corrected hypo[Cl^−^] was higher than those with borderline (OR: 5.74 [95% CI: 4.09–8.05]; *p* < 0.0001) or mild (OR: 4.80 [95% CI: 2.62–8.96]; *p* < 0.0001) prehospital corrected hypo[Cl^−^], and the case‐fatality rate of cats with hospital‐acquired corrected hypo[Cl^−^] was higher than those with borderline prehospital corrected hypo[Cl^−^] (OR: 3.09 [95% CI: 2.0–4.78]; *p* < 0.0001) (Tables 2 and 3).

**TABLE 2:** Prehospital versus hospital‐acquired corrected hypochloremia in 2388 dogs with corrected hypochloremia.

	Prehospital (*n* = 1779)			Hospital‐acquired (*n* = 498)		
	Nonsurvivors	Survivors	Fatality	Nonsurvivors	Survivors	Fatality				
	(*n*)	(*n*)	(%)	(*n*)	(*n*)	(%)	*p*‐value	OR	95% CI	
Borderline (*n* = 1642)	66	1120	5.6	96	283	25.3	<0.0001	5.74	4.09	8.05
Mild (*n* = 495)	30	363	7.6	23	58	28.4	<0.0001	4.80	2.62	8.96
Moderate (*n* = 149)	20	95	17.4	7	23	23.3	0.44	1.45	0.57	3.94
Severe (*n* = 102)	21	64	24.7	2	6	25.0	>0.99	1.02	0.20	4.54
Total (*n* = 2388)	137	1642	7.7	128	370	25.7	<0.0001	4.15	3.18	6.76

*Note*: Of these 2388 dogs, 1779 were categorized as having prehospital corrected hypochloremia, while 498 were categorized as having hospital‐acquired corrected hypochloremia. Cases with an unknown immediate prehospital history (111 dogs) are included in the total. Dogs with blood or serum corrected [Cl^−^] lower than the reference intervals were identified and categorized as severe (≥10 mmol/L lower than the low end of the reference interval), moderate (7–9 mmol/L lower than the low end of the reference interval), mild (4–6 mmol/L lower than the low end of the reference interval), or borderline (≤3 mmol/L lower than the low end of the reference interval) corrected hypo[Cl^−^]. The *p*‐values were obtained by comparing the fatality rates of prehospital corrected hypo[Cl^−^] and hospital‐acquired corrected hypo[Cl^−^] in different severities of corrected hypo[Cl^−^].

Abbreviations: CI, confidence interval; OR, odds ratio.

**TABLE 3:** Prehospital versus hospital‐acquired corrected hypochloremia in 1463 cats with corrected hypochloremia.

	Prehospital (*n* = 1091)			Hospital‐acquired (*n* = 253)						
	Nonsurvivors	Survivors	Fatality	Nonsurvivors	Survivors	Fatality				
	(*n*)	(*n*)	(%)	(*n*)	(*n*)	(%)	*p*‐value	OR	95% CI	
Borderline (*n* = 961)	58	637	8.4	41	146	21.9	<0.0001	3.09	2.0	4.78
Mild (*n* = 294)	55	174	24.0	13	30	30.2	0.44	1.37	0.69	2.72
Moderate (*n* = 112)	15	72	17.2	3	14	17.6	>0.99	1.03	0.29	3.98
Severe (*n* = 96)	15	65	18.8	2	4	33.3	0.34	2.17	0.38	9.94
Total (*n* = 1463)	143	948	13.1	59	194	23.3	<0.0001	2.02	1.44	2.84

*Note*: Of these 1463 cats, 1091 were categorized as having prehospital corrected hypochloremia, while 253 were categorized as having hospital‐acquired corrected hypochloremia. Cases with unknown outcomes (119 cats) are included in the total. Cats with blood or serum corrected [Cl^−^] lower than the reference intervals were identified and categorized as severe (≥10 mmol/L lower than the low end of the reference interval), moderate (7–9 mmol/L lower than the low end of the reference interval), mild (4–6 mmol/L lower than the low end of the reference interval), or borderline (≤3 mmol/L lower than the low end of the reference interval) corrected hypo[Cl^−^]. The *p*‐values were obtained by comparing the fatality rates of prehospital corrected hypo[Cl^−^] and hospital‐acquired corrected hypo[Cl^−^] in different severities of corrected hypo[Cl^−^].

Abbreviations: CI, confidence interval; OR, odds ratio.

### 3.5 The Primary Disease Processes Associated With Corrected Hypo[Cl^−^]

The most common primary disease processes identified in dogs with mild, moderate, or severe corrected hypo[Cl^−^] were urologic (224/746 [30.0%]), cardiovascular (192/746 [25.7%]), and gastrointestinal diseases (176/746 [23.6%]) (Table 4). In cats, the most frequently reported primary disease processes were urologic (255/502 [50.8%]), cardiovascular (155/502 [30.9%]), and gastrointestinal diseases (115/502 [22.9%]) (Table 5). The proportion of animals with corrected hypo[Cl^−^] over those with normal corrected [Cl^−^] with each primary disease process was also investigated (Tables 6 and 7). In dogs, corrected hypo[Cl^−^] was noted in 31.6% (81/256) cases with pancreatic disease, 30.3% (44/145) cases with DM/DKA, and 23.7% (192/811) cases with cardiovascular disease (Table 6). In cats, corrected hypo[Cl^−^] was observed in 64.7% (55/85) cases with DM/DKA, 38.7% (53/137) cases with pancreatic disease, and 35.8% (155/433) cases with cardiovascular disease (Table 7). Applying Bonferroni correction found a significant association between the various primary disease processes and the documentation of corrected hypo[Cl^−^]. These primary disease processes included pancreatic, DM/DKA, cardiovascular, hyperadrenocorticism, respiratory, hematologic, and gastrointestinal diseases in dogs (Table 6), and DM/DKA, pancreatic, cardiovascular, hepatobiliary, and urologic diseases in cats (Table 7).

**TABLE 4:** Primary disease processes of 746 dogs with mild (495 dogs), moderate (149), or severe (102) corrected hypochloremia presented to a referral veterinary hospital.

	Mild (*n*)	Moderate (*n*)	Severe (*n*)	Total (*n*)	Percent (%)
Urologic	146	44	34	224	30.0
Cardiovascular	121	53	18	192	25.7
Gastrointestinal	107	34	35	176	23.6
Respiratory	105	38	13	156	20.9
Neoplastic	103	36	16	155	20.8
Neurologic	96	27	17	140	18.8
Hepatobiliary	15	23	61	99	13.3
Pancreatic	19	24	38	81	10.9
Hematologic	5	17	50	72	9.7
Oropharyngeal	3	9	37	49	6.6
DM/DKA	12	10	22	44	5.9
Musculoskeletal	3	7	29	39	5.2
Hyperadrenocorticism	3	12	20	35	4.7
Septic	3	3	13	19	2.5
Hypothyroidism	1	4	10	15	2.0
Hypoadrenocorticism	3	5	7	12	1.6
Toxicoses	5	6	0	11	1.5
CPA	2	1	4	7	0.9
Reproductive	2	1	3	6	0.8
Diabetes insipidus	0	0	1	1	0.1
Hyperparathyroidism	0	0	0	0	0

*Note*: Individual animals may have more than one condition. Dogs with blood or serum corrected [Cl^−^] lower than the reference intervals were identified and categorized as severe (≥10 mmol/L lower than the low end of the reference interval), moderate (7–9 mmol/L lower than the low end of the reference interval), or mild (4–6 mmol/L lower than the low end of the reference interval) corrected hypo[Cl^−^].

Abbreviations: CPA, cardiopulmonary arrest; DM/DKA, diabetes mellitus/diabetic ketoacidosis.

**TABLE 5:** Primary disease processes of 502 cats with mild (294 cats), moderate (112), or severe (96) corrected hypochloremia presented to a referral veterinary hospital.

	Mild (*n*)	Moderate (*n*)	Severe (*n*)	Total (*n*)	Percent (%)
Urologic	150	56	49	255	50.8
Cardiovascular	80	35	40	155	30.9
Gastrointestinal	60	26	29	115	22.9
Neoplastic	53	28	17	98	19.5
Respiratory	50	18	17	85	16.9
Hepatobiliary	50	16	12	78	15.5
Hematologic	33	13	10	56	11.2
DM/DKA	26	14	14	55	11.0
Pancreatic	28	10	15	53	10.7
Hyperthyroidism	24	9	5	38	7.6
Oropharyngeal	24	6	3	33	6.6
Hypothyroidism	2	0	0	2	0.4
Neurologic	21	0	0	21	4.2
Musculoskeletal	8	0	1	9	1.8
Septic	6	0	2	8	1.6
Toxicoses	2	0	0	2	0.4
CPA	2	0	0	2	0.4
Hyperadrenocorticism	1	0	0	1	0.2
Reproductive	1	0	0	1	0.2
Hypoadrenocorticism	0	0	0	0	0
Diabetes insipidus	0	0	0	0	0

*Note*: Individual animals may have more than one condition. Cats with blood or serum corrected [Cl^−^] lower than the reference intervals were identified and categorized as severe (≥10 mmol/L lower than the low end of the reference interval), moderate (7–9 mmol/L lower than the low end of the reference interval), or mild (4–6 mmol/L lower than the low end of the reference interval) corrected hypo[Cl^−^].

Abbreviations: CPA, cardiopulmonary arrest; DM/DKA, diabetes mellitus/diabetic ketoacidosis.

**TABLE 6:** The proportion of cases with mild (495 dogs), moderate (149), and severe (102) corrected hypochloremia versus normal corrected chloride (11,125 dogs) with each primary disease process in a total of 11,871 dogs.

Disease processes	Corrected hypo[Cl^−^] (*n*)	Total # of corrected [Cl^−^] (*n*)	Proportions of hypo[Cl^−^] (%)	OR	*p*‐value	95% CI	
**Pancreatic**	**81**	**256**	**31.6**	**7.64**	**<0.0001**	**5.81**	**10.05**
**DM/DKA**	**44**	**145**	**30.3**	**6.88**	**<0.0001**	**4.80**	**9.87**
**Cardiovascular**	**192**	**811**	**23.7**	**5.88**	**<0.0001**	**4.89**	**7.06**
**CPA**	**7**	**31**	**22.6**	**4.60**	**0.0002**	**2.02**	**10.45**
**Hyperadrenocorticism**	**35**	**194**	**18.0**	**3.43**	**<0.0001**	**2.37**	**4.97**
**Respiratory**	**156**	**970**	**16.1**	**3.35**	**<0.0001**	**2.76**	**4.05**
**Sepsis**	**19**	**118**	**16.1**	**2.97**	**<0.0001**	**1.82**	**4.85**
**Hypoadrenocorticism**	**12**	**80**	**15.0**	**2.75**	**0.0013**	**1.5**	**5.04**
**Hematologic**	**72**	**496**	**14.5**	**2.71**	**<0.0001**	**2.09**	**3.52**
**Gastrointestinal**	**176**	**1388**	**12.7**	**2.53**	**<0.0001**	**2.12**	**3.03**
**Hepatobiliary**	**99**	**922**	**10.7**	**1.92**	**<0.0001**	**1.53**	**2.40**
**Urologic**	**224**	**2063**	**10.9**	**1.84**	**<0.0001**	**1.84**	**2.55**
Diabetes insipidus	1	12	8.3	1.95	0.54	0.35	10.68
Hypothyroidism	15	229	6.6	1.08	0.87	0.64	1.82
Toxicoses	11	190	5.8	0.95	0.95	0.52	1.74
Neurology	140	2714	5.2	0.77	0.0059	0.64	0.93
Neoplasia	155	3029	5.1	0.75	0.0022	0.63	0.90
Reproductive	6	177	3.4	0.56	0.11	0.26	1.23
**Oropharyngeal**	**49**	**1519**	**3.2**	**0.46**	**<0.0001**	**0.34**	**0.62**
**Musculoskeletal**	**39**	**1388**	**2.8**	**0.40**	**<0.0001**	**0.29**	**0.55**

*Note*: Dogs could have more than one primary disease process. The *p*‐values were obtained by comparing the proportion of normal corrected [Cl^−^] and corrected hypo[Cl^−^] among the total number of dogs with each primary disease process. After Bonferroni correction, *p* < 0.002 was considered statistically significant and such rows are presented in **bold font**.

Abbreviations: CI, confidence interval; CPA, cardiopulmonary arrest; DM/DKA, diabetes mellitus/diabetic ketoacidosis; OR, odds ratio.

**TABLE 7:** The proportion of cases with mild (294 cats), moderate (112), and severe (96) corrected hypochloremia versus normal corrected chloride (2350 cats) with each primary disease process in a total of 2852 cats.

Disease processes	Corrected hypo[Cl^−^] (*n*)	Total # of corrected [Cl^−^] (*n*)	Proportions of hypo[Cl^−^] (%)	OR	*p*‐value	95% CI	
**DM/DKA**	**55**	**85**	**64.7**	**9.45**	**<0.0001**	**6.00**	**14.85**
**Pancreatic**	**53**	**137**	**38.7**	**3.18**	**<0.0001**	**2.23**	**4.55**
**Cardiovascular**	**155**	**433**	**35.8**	**3.33**	**<0.0001**	**2.66**	**4.17**
**Hepatobiliary**	**78**	**213**	**36.6**	**3.02**	**<0.0001**	**2.24**	**4.04**
CPA	2	6	33.3	2.61	0.29	055	12.26
**Urologic**	**255**	**830**	**30.7**	**3.19**	**<0.0001**	**2.61**	**3.88**
**Respiratory**	**85**	**285**	**29.8**	**2.19**	**<0.0001**	**1.66**	**2.88**
**Hematologic**	**56**	**204**	**27.5**	**1.87**	**0.0001**	**1.35**	**2.58**
**Gastrointestinal**	**115**	**491**	**23.4**	**1.56**	**0.0002**	**1.24**	**1.98**
Hyperthyroidism	38	204	18.6	1.08	0.69	0.74	1.55
Sepsis	8	46	17.4	0.99	0.97	0.47	2.07
Neoplasia	98	697	14.1	0.71	0.0047	0.56	0.90
Neurology	21	200	10.5	0.53	0.062	0.33	0.83
Reproductive	1	10	10.0	0.74	>0.99	0.13	4.14
**Oropharyngeal**	**33**	**506**	**6.5**	**0.28**	**<0.0001**	**0.19**	**0.40**
Toxicoses	2	37	5.4	0.33	0.050	0.09	1.18
**Musculoskeletal**	**9**	**165**	**5.6**	**0.26**	**<0.0001**	**0.14**	**0.50**

*Note*: Cats could have more than one disease process. The *p*‐values were obtained by comparing the proportion of normal corrected [Cl^−^] and corrected hypo[Cl^−^] among the total number of cats with each primary disease process. After Bonferroni correction, *p* < 0.002 was considered statistically significant and such rows are presented in **bold font**.

Abbreviations: CI, confidence interval; CPA, cardiopulmonary arrest; DM/DKA, diabetes mellitus/diabetic ketoacidosis; OR, odds ratio.

## 4 Discussion

Both measured and corrected hypo[Cl^−^] were common electrolyte abnormalities in dogs and cats visiting the tertiary veterinary hospital. In human medicine, prevalence of measured hypo[Cl^−^] was reported to range from 8.8% to 37% in the general ICU [3, 12]. In the veterinary literature, one study reported that the prevalence of measured hypo[Cl^−^] in dogs and cats at a veterinary teaching hospital was 19.6% and 44.3%, respectively [17]. In cats, a previous study at a veterinary teaching hospital found the prevalence of measured and corrected hypo[Cl^−^] to be 27% and 19%, respectively [9]. In the current study, the prevalence of measured hypo[Cl^−^] was 23.3% in dogs and 59.0% in cats, while the prevalence of corrected hypo[Cl^−^] was 13.9% and 34.9% in dogs and cats, respectively. The difference in the prevalence of hypo[Cl^−^] in these studies could be due to different study settings and populations. The current study included all dogs and cats with at least one measurement of [Cl^−^] and [Na^+^] admitted to the teaching hospital, encompassing not only those admitted due to systemic illness but also those presented for annual examination in the primary care service. Therefore, the varying severity of illness and underlying disease processes in these animals compared with those in the previous study may partially explain the differences in the prevalence of hypo[Cl^−^].

Chloride ions are the most abundant anions in the body and play a crucial role in maintaining cellular and tissue homeostasis. Their depletion may result in a wide range of abnormalities, contributing to the higher mortality rate. Several studies reported that abnormal water balance and [Na^+^] negatively affect both [Cl^−^] and the outcome in human and veterinary patients [25, 26, 27]. In the current study, case‐fatality rates of both dogs and cats with measured and corrected hypo[Cl^−^] were higher than those with normal measured and corrected [Cl^−^]. This relationship was retained even in dogs and cats with borderline measured and corrected hypo[Cl^−^]. This indicates that either direct depletion of chloride ions with a relative decrease in [Cl^−^] compared with [Na^+^] or an abnormal water balance causing a proportional reduction in both [Cl^−^] and [Na^+^] may be associated with a higher mortality rate. Moreover, cats with corrected hypo[Cl^−^] in the current study showed a higher case‐fatality rate than those with measured hypo[Cl^−^], raising the suspicion that chloride ion depletion may have a greater impact on mortality than alterations in water balance, especially in cats.

The case‐fatality rate also linearly increased as the severity of measured and corrected hypo[Cl^−^] worsened, except in cats with corrected hypo[Cl^−^]. A similar relationship has been reported in people, dogs, and cats with measured hypo[Cl^−^] [20, 21, 28, 29]. However, there are no linear correlations between corrected hypo[Cl^−^] and mortality in these previous studies in dogs and cats, presumably due to lower case numbers [20, 21]. The reason for higher case‐fatality rates in cats with mild corrected [Cl^−^] than those with moderate or severe corrected hypo[Cl^−^] was not determined, but it may be due to a higher rate of euthanasia in this group of cats. Notably, a previous study found no association between the case‐fatality rate and corrected [Cl^−^] in cats when euthanized animals were excluded from the analysis [21]. Due to the retrospective nature of the current study, the cause of death was not fully investigated, and the relationship between hypo[Cl^−^] and mortality associations may have differed if euthanized animals had been excluded from the nonsurvivor group.

Although acid–base balance was not assessed in this study due to limited data, it is speculated that corrected hypo[Cl^−^] is associated with the development of metabolic alkalosis [1, 2, 30, 31]. In human patients, altered mentation, seizures, cardiac arrhythmias, myoclonus, and tetany are clinical signs of hypochloremic metabolic alkalosis [2]. Although clinical signs directly associated with corrected hypo[Cl^−^] due to pure chloride loss have not been extensively investigated in animals, metabolic alkalosis associated with corrected hypo[Cl^−^] is considered a potential contributor. Indeed, metabolic alkalosis is the most common acid–base abnormality identified in animals with hypo[Cl^−^] [9, 17, 32]. In cats, generalized muscle fasciculation or tremors have been reported with gastrointestinal obstruction, and Jukes et al. attributed the clinical signs to severe hypochloremic metabolic alkalosis with measured [Cl^−^] of 74 mmol/L [30]. However, such clinical signs are not expected in animals with mild hypo[Cl^−^]. Nevertheless, the exact mechanisms behind these clinical signs in animals with severe hypo[Cl^−^] remain undetermined, and the severity of corrected hypo[Cl^−^] may simply reflect the severity of the underlying diseases [33].

The majority of dogs and cats with documented corrected hypo[Cl^−^] had prehospital hypo[Cl^−^] (74.5% in dogs and 74.6% in cats), which aligns with reports in human patients and suggests that corrected hypo[Cl^−^] is associated with underlying disorders [12, 34]. Notably, higher case‐fatality rates were seen in both dogs and cats with hospital‐acquired corrected hypo[Cl^−^] compared with those with corrected hypo[Cl^−^] diagnosed at presentation. This observation may be attributed to the worse prognosis in animals with progressive underlying diseases that did not respond to the medical intervention or with iatrogenic causes of corrected hypo[Cl^−^], contributing to the development of corrected hypo[Cl^−^] during hospitalization. However, it is important to acknowledge that a direct contribution of corrected hypo[Cl^−^] to increased mortality cannot be ruled out. In addition, the cause of death (euthanasia or natural death), which was not investigated in this study, could influence these outcomes.

Among the primary disease processes associated with corrected hypo[Cl^−^], urologic disease was the most common in both dogs (30.0%) and cats (50.8%) (Tables 4 and 5). In cases of corrected hypo[Cl^−^], excess chloride ions can be lost through the gastrointestinal system via vomiting and diarrhea or lost into the urine, leading to hypo[Cl^−^] not explained by changes in water balance. Additionally, animals with severe uremic acidosis are sometimes treated with sodium bicarbonate, which would contribute to the development or exacerbation of corrected hypo[Cl^−^] [2]. It is also possible that these animals with urologic disease had another comorbidity, such as cardiac disease, and were treated with diuretics, leading to the development of corrected hypo[Cl^−^] [9, 10]. Nevertheless, it is important to note that the majority of dogs and cats with urologic disease had normal corrected [Cl^−^], and 10.9% of dogs and 30.7% of cats with urologic disease had corrected hypo[Cl^−^] (Tables 6 and 7). Therefore, it is unknown if the urologic disease is the cause of these animals developing corrected hypo[Cl^−^] or if there were other contributing factors, such as iatrogenic causes.

Cardiovascular disease was the second most common primary disease process associated with corrected hypo[Cl^−^] in dogs (25.7%) and cats (30.9%) (Tables 4 and 5). Within the group of animals with cardiovascular disease, 23.7% of dogs and 35.8% of cats were diagnosed with corrected hypo[Cl^−^] (Tables 6 and 7). Underlying cardiac disease and diuretic administration are recognized as potential contributors to corrected hypo[Cl^−^] in patients with cardiovascular disease, particularly in cases of CHF [4, 13, 35, 36]. In dogs and cats, recent retrospective studies demonstrated that both measured and corrected hypo[Cl^−^] were observed in a substantial proportion of animals with acute CHF at admission, and a significant negative correlation was found between chloride concentrations at admission and furosemide doses at discharge in dogs [8, 10]. One of these studies also reported lower corrected [Cl^−^] in dogs with CHF refractory to diuretic therapy compared to dogs with CHF that responded to diuretics [10]. These findings suggest that corrected hypo[Cl^−^] could be associated with the severity of cardiovascular disease, especially CHF, and corrected hypo[Cl^−^] could indicate refractory CHF requiring higher doses of diuretics and carrying a poorer prognosis [19, 35]. Furosemide, a loop diuretic, inhibits Na:K:2Cl^−^ cotransporters, and high doses of furosemide result in corrected hypo[Cl^−^] due to the disproportionate loss of chloride ions compared with sodium ions [37]. Also, chloride ions are involved in the function of the tubuloglomerular feedback system, which mediates the vasomotor tone of afferent arterioles and the renin–angiotensin–aldosterone system [38, 39]. Adequate chloride concentration suppresses the release of renin, while hypo[Cl^−^] stimulates its release. Therefore, correcting hypo[Cl^−^] by supplementing chloride ions may be considered as a novel therapeutic approach for refractory CHF [10, 35]. Finally, severe hypo[Cl^−^] has been reported to be associated with decreased left ventricular ejection fraction, increased cardiac function markers, and circulating catecholamine, which could contribute to poor outcomes in patients with cardiovascular disease in conjunction with hypo[Cl^−^] [4, 18, 40].

Gastrointestinal disease was the third most common disease process associated with corrected hypo[Cl^−^] in dogs (23.6%) and cats (22.9%). In dogs and cats with gastrointestinal disease, 12.7% of dogs and 23.4% of cats had corrected hypo[Cl^−^]. This occurrence can be attributed to gastrointestinal chloride loss through vomiting, diarrhea, or aspiration of nasogastric tubes. Previous research has also shown a high prevalence of corrected hypo[Cl^−^] in cats presenting with vomiting as the most common clinical sign [9]. Hypo[Cl^−^] has also been identified as the most common electrolyte abnormality in dogs with gastrointestinal foreign body obstructions [7]. However, a recent study showed that the incidence of metabolic alkalosis in upper gastrointestinal obstruction is lower than previously reported [41]. Another recent prospective study did not find an association between intermittent nasogastric tube aspiration and the development of corrected hypo[Cl^−^] in dogs and cats [42].

Although these disease processes did not represent the most common causes of corrected hypo[Cl^−^] in dogs and cats, animals with DM/DKA (5.9% in dogs and 11.0% in cats) and pancreatic disease (10.9% in dogs and 10.7% in cats) encompassed the largest proportion of cases that were also diagnosed with corrected hypo[Cl^−^]. This finding could be attributable to the frequent development of gastrointestinal signs, including vomiting and diarrhea, in animals with DM/DKA and pancreatic diseases, leading to the depletion of chloride ions [43, 44, 45]. However, the proportion of animals with corrected hypo[Cl^−^] in DM/DKA (31.6% in dogs and 64.7% in cats) and pancreatic disease (30.3% in dogs and 38.7% in cats) was largely different from that of those with gastrointestinal diseases. Therefore, the development of corrected hypo[Cl^−^] in animals with DM/DKA and pancreatic disease may not be explained solely by the loss of chloride ions into the gastrointestinal system.

Several inherent limitations should be considered when interpreting the findings of this retrospective study. Although standard institutional procedures for handling samples were established at the tertiary referral hospital where the samples were collected, adherence to these protocols cannot be guaranteed due to the retrospective study design. Because records often do not state the clear reason for euthanasia, causes of death, including both natural death and euthanasia, were grouped together. Excluding cases of euthanasia might change the observed association between hypo[Cl^−^] and case‐fatality rate. Second, limited data on the timing of chloride concentration measurements in relation to therapeutic interventions made it challenging to determine the precise effects of medical interventions on chloride and sodium concentrations. This limitation is relevant for both prehospital and hospital‐acquired corrected hypo[Cl^−^]. Hospital‐acquired corrected hypo[Cl^−^] was diagnosed based on the timing of the documentation rather than the identification of iatrogenic causes. Due to the retrospective study design, resuscitation therapy might have been initiated before obtaining the initial blood sample at presentation in a small proportion of cases. Further studies are warranted to investigate whether certain fluid choices, medications, or procedures are associated with the development of hypo[Cl^−^]. In addition, acid–base status was not reported for the animals included in the current study, because this information was not available from the serum biochemistry profiles. Previous studies have indicated that a considerable percentage of dogs and cats with metabolic alkalosis were diagnosed with corrected hypo[Cl^−^], highlighting the need to evaluate the role played by hypo[Cl^−^] in the context of the animal's acid–base status [32, 46]. In this study, the primary disease processes associated with corrected hypo[Cl^−^] were determined based on the primary clinicians’ final diagnoses. Although these underlying diseases were likely associated with corrected hypo[Cl^−^], they do not necessarily represent the primary etiology or pathophysiological mechanisms leading to corrected hypo[Cl^−^]. In addition, chloride concentrations could have been influenced by specific therapies, including hemodialysis and intravenous fluid administration. Due to the retrospective nature of the study, the potential impact of these therapies on electrolyte concentrations was not analyzed. As a result, the etiologies of hospital‐acquired corrected hypo[Cl^−^] were not assessed. Finally, because this study was conducted at a single tertiary referral hospital and included both systemically healthy and ill animals, the true prevalence of corrected hypo[Cl^−^] in systemically ill dogs and cats could not be determined. Future multicenter prospective studies are warranted to determine the true prevalence of corrected hypo[Cl^−^] and its associations with underlying diseases and mortality. In addition, systemic illness should be objectively assessed using a validated scoring system, and further studies should also consider comparing the systemic illness score with the development of hypo[Cl^−^].

In conclusion, animals with measured and corrected hypo[Cl^−^] had higher case‐fatality rates compared with those with normal measured and corrected [Cl^−^], and the severity of measured and corrected hypo[Cl^−^] demonstrated a linear association with case‐fatality rate. The majority of corrected hypo[Cl^−^] cases were diagnosed at admission rather than developing during hospitalization. Animals with hospital‐acquired corrected hypo[Cl^−^] had higher case‐fatality rates compared with those with prehospital corrected hypo[Cl^−^]. The common primary disease processes reported in animals with corrected hypo[Cl^−^] include urologic, cardiovascular, and gastrointestinal diseases. Further prospective studies are needed to determine whether abnormal corrected [Cl^−^] directly contributes to morbidity or serves as a marker of disease severity in dogs and cats.

## Author Contributions

**Yu Ueda**: conceptualization, data curation, formal analysis, investigation, methodology, project administration, resources, software, validation, visualization, writing – original draft. **Steven E. Epstein**: conceptualization, formal analysis, investigation, methodology, project administration, resources, software, supervision, validation, writing – review and editing. **Kate Hopper**: conceptualization, investigation, methodology, project administration, resources, software, supervision, validation, writing – review and editing.

## Conflicts of Interest

Dr. Epstein is an Assistant Editor of the Journal but only participated in the peer review process as an author. The authors declare no other conflicts of interest.

## Endnotes

1 ABL 705, Radiometer Medical A/S, Copenhagen, Denmark.

2 Chemistry Analyzer, Hitachi 917 and Hitachi c501, Roche Diagnostics, Indianapolis, IN.

3 GraphPad Software Prism Version 9.3.1, San Diego, CA.
